# Midbody: From the Regulator of Cytokinesis to Postmitotic Signaling Organelle

**DOI:** 10.3390/medicina54040053

**Published:** 2018-07-30

**Authors:** Ieva Antanavičiūtė, Paulius Gibieža, Rytis Prekeris, Vytenis Arvydas Skeberdis

**Affiliations:** 1 Institute of Cardiology, Medical Academy, Lithuanian University of Health Sciences, LT-50162 Kaunas, Lithuania; Ieva.Antanaviciute@lsmuni.lt (I.A.); Paulius.Gibieza@lsmuni.lt (P.G.); 2 Department of Cell and Developmental Biology, University of Colorado Anschutz Medical Campus, Aurora, CO 80045, USA; Rytis.Prekeris@ucdenver.edu

**Keywords:** cytokinesis, intercellular bridge, midbody, conductance, permeability, tumorigenicity

## Abstract

Faithful cell division is crucial for successful proliferation, differentiation, and development of cells, tissue homeostasis, and preservation of genomic integrity. Cytokinesis is a terminal stage of cell division, leaving two genetically identical daughter cells connected by an intercellular bridge (ICB) containing the midbody (MB), a large protein-rich organelle, in the middle. Cell division may result in asymmetric or symmetric abscission of the ICB. In the first case, the ICB is severed on the one side of the MB, and the MB is inherited by the opposite daughter cell. In the second case, the MB is cut from both sides, expelled into the extracellular space, and later it can be engulfed by surrounding cells. Cells with lower autophagic activity, such as stem cells and cancer stem cells, are inclined to accumulate MBs. Inherited MBs affect cell polarity, modulate intra- and intercellular communication, enhance pluripotency of stem cells, and increase tumorigenic potential of cancer cells. In this review, we briefly summarize the latest knowledge on MB formation, inheritance, degradation, and function, and in addition, present and discuss our recent findings on the electrical and chemical communication of cells connected through the MB-containing ICB.

## 1. Introduction

**Figure 1:**
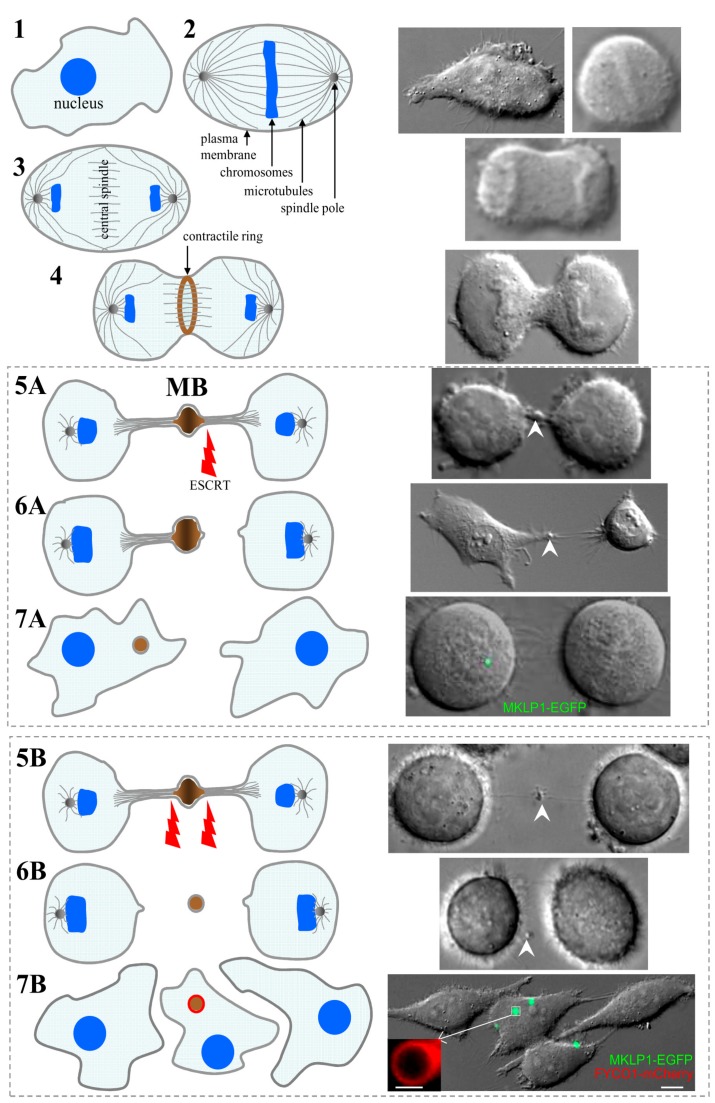
Midbody (MB) formation and inheritance. Schematic views on the left panel and photos on the right panel represent dividing HeLa cells expressing MKLP1-EGFP, a well-established MB marker. 1—cells at the end of interphase; 2—during metaphase, duplicated chromosomes accumulate at the midzone; 3—during anaphase, two sets of chromatids translocate to the opposing spindle poles; 4—the actomyosin contractile ring constricts the cell forming an ingression of the cleavage furrow; 5—further constriction of the contractile ring results in formation of the intercellular bridge (ICB) (membrane scission on one (A) or both (B) sides of MB is catalyzed by endosomal sorting complex required for transport (ESCRT)); 6—abscission occurs on either one (A—asymmetric abscission) or both (B—symmetric abscission) sides of the MB; 7—MB can be inherited by one of the daughter cells (A) or released into the extracellular space and later engulfed by the same or other surrounding cells (B). The scale bars in 7B and its inset indicate 10 µm and 1 µm, respectively.

Mitotic cell division is one of the most fundamental aspects of cell life and is a key event during organogenesis as well as tissue maintenance and function. The failure to faithfully divide the genomic material as well as cytoplasmic organelles and signaling molecules often lead to severe consequences such as cancerogenesis and various developmental disorders. Cytokinesis is a terminal stage of cell division leaving two daughter cells connected through the intercellular bridge (ICB). During cytokinesis, central spindle microtubules are packed into a structure named the midbody (MB) [1] that is situated within the ICB. Cytokinesis completes with the final resolution of the ICB, known as abscission, resulting in physical separation of two daughter cells. Intriguingly, the dense network of microtubules within the MB prevents the abscission occurring at the MB. Instead, the abscission occurs on one or both sides of the MB (Figure 1). Consequently, there are two possible outcomes of cytokinesis: asymmetric and symmetric ICB abscission (Figure 1). In the first case, the MB can be incorporated by one of the daughter cells, while during symmetric ICB abscission, the MB is released into the extracellular milieu and later may be engulfed by the surrounding cells. The MB was commonly regarded as a structure that regulates the abscission. Thus, it was assumed that upon completion of the mitotic division, the postmitotic MBs were rapidly degraded. However, recent studies have demonstrated that postmitotic MBs can actually be retained by cells for prolonged periods (often referred to as MB inheritance). Significantly, it has been shown that inherited postmitotic MBs can affect cell polarity, modulate intracellular signaling and intercellular communication, enhance pluripotency of stem cells, and increase tumorigenic potential of cancer cells. In this review, we summarize the newest findings regarding MB formation and inheritance as well as present our own new data on the conductance and permeability of the MB-containing ICB that have not been sufficiently described in the scientific literature yet.

## 2. Midbody Formation and Inheritance

During metaphase, duplicated chromosomes accumulate at the midzone of the dividing cell (Figure 1, step 2). Cytokinesis starts during anaphase and is the final step of cell division. During anaphase, two sets of chromatids translocate to the opposing spindle poles (step 3). Overlapping microtubules of the mitotic spindle compose the central spindle, and the further assembly of it is regulated by the heterotetrameric centralspindlin complex, which is composed of two subunits of MKLP1, a kinesin-6 motor protein, and CYK-4, a Rho-family GTPase-activating protein [2]. Activated RhoA coordinates the assemblage of the actomyosin contractile ring, which constricts the cell forming an ingression of the cleavage furrow (step 4). The contractile ring is built of formin-nucleated actin filaments, bipolar filaments composed of the motor, myosin II, membrane-associated septin filaments, and anillin cross-linking the actin filaments [3]. The further constriction of the contractile ring results in a formation of the ICB, which connects two daughter cells (step 5). The ICB is 1–3 µm in thickness and contains residuals of the contractile ring and central spindle microtubules situated in anti-parallel overlapping bundles. The overlapping plus-ends of these microtubules shape the MB, a structure located at the center of the ICB (Video S1). More than 100 years ago, Dr. Walter Flemming was the first who noticed and described this structure, which was then named after him (Flemming body), recently known as the midbody (MB) [4]. Due to the density of the overlapping MB microtubules, the abscission can never occur at the MB. Instead, the abscission occurs on either one (asymmetric abscission) or both (symmetric abscission) sides of the MB (steps 6A and 6B, respectively). It is now well established that determination of the abscission site is a highly regulated event that is determined by local changes in actin, microtubule, and endosome dynamics [5].

Abscission is a very complicated event since it involves coordinated depolymerization of ICB actin cytoskeleton, severing of central spindle microtubules, and final fusion of the plasma membrane. The molecular machinery that determines the abscission site and governs localized changes in the actin and microtubule cytoskeleton is only beginning to be determined; however, it is now clear that abscission starts by localized depolymerization of the actin cytoskeleton. The actin cytoskeleton enriched at the ICB is the remnant of the actomyosin contractile ring and was suggested to inhibit microtubule severing and plasma membrane fusion [6]. Thus, localized clearing of the actin cytoskeleton likely serves as a determinant of the future abscission site. How spatiotemporal actin dynamics within the ICB is regulated remains to be fully understood; however, actin depolymerization and abscission initiation have been shown to depend on Rab11- and Rab35-containing endosomes that are transported to the ICB just before the abscission event [7,8]. Rab11-endosomes deliver p50RhoGAP [9] while Rab35-endosomes deliver OCRL and MICAL, all proteins that lead to localized depolymerization of the actin cytoskeleton and are required for abscission [7,10]. On removal of the actin cytoskeleton from the ICB, the cell enters the final stage of the abscission, the severing central spindle microtubules and fusion of the ICB plasma membrane. This abscission step relays on the endosomal sorting complex required for transport (ESCRT) [11]. The ESCRTs were originally identified as proteins needed for the generation of intraluminal vesicles during lysosomal protein degradation and maturation of late endosomes [11]. While there are four separate ESCRTs (ESCRT 0, I, II, and III), only ESCRT-III generates oligomeric structures that are thought to drive membrane bending and eventual fusion. Consistently, only the ESCRT-III has been shown to form oligomeric spiral structures at the abscission site, and has been known to be required for cytokinesis [12,13]. Importantly, ESCRT-III is first recruited to the MB, presumably by binding to the ALIX/CEP55 complex. Just before abscission, ESCRT-III re-localizes to the abscission site, where it mediates further ICB thinning and eventual resolution of the ICB. In addition to plasma membrane fusion, ESCRTs also play a role in microtubule severing. It has been shown that microtubule-severing protein, such as spastin, can bind directly to the ESCRT-III [11,14], presumably ensuring the coordination of microtubule depolymerization and plasma membrane fusion at the abscission site (Figure 1). What remains unclear is why ESCRT-III first accumulates at the MB and what mechanism drives ESCRT-III translocation from the MB to the abscission site. At least two competing (but not necessarily mutually exclusive) models have been proposed. The first model suggests that ESCRT-III oligomers initiate at the MB and then spiral toward the abscission site thus gradually narrowing the ICB [12]. An alternative model proposes that the MB is simply an ESCRT-III “staging/activation station” to allow the accumulation of ESCRTs in the diffusion-limited ICB. This model suggests that on the establishment of the abscission site by actin depolymerization, ESCRT-III can then form de novo spirals at the abscission site [9]. Further research will be needed to define ESCRT-III-dependent abscission as well as to dissect the machinery regulating this process.

## 3. The Roles of Postmitotic MBs in Regulating Cell Polarity and Fate

Multiple studies from several laboratories have clearly established that abscission is a very complex and highly-regulated event. It was shown that ESCRT-III can catalyze membrane scission on one (asymmetric abscission) or on both (symmetric abscission) sides of the MB to separate the two daughter cells (Figure 1, step 5). In the first case, the MB is asymmetrically inherited by one of the daughter cells (steps 6A and 7A) (Video S2) [5]. In the second case, the MB is released into the extracellular space (Video S3) and later may be engulfed by the same or other surrounding cells (steps 6B and 7B) [15]. Interestingly, it has been shown that stem cells and highly aggressive cancer cells are the ones that tend to undergo asymmetric abscission leading to the cytoplasmic accumulation of postmitotic MBs [1,16]. Thus, it has been proposed that postmitotic MBs function as a specialized signaling platform regulating cell stemness and differentiation. However, this suggestion remains highly controversial and further studies will be needed to define the role of postmitotic MBs in regulating cell stemness.

While the involvement of postmitotic MBs in regulating cell stemness remains to be confirmed, the role of MBs in establishing cell polarity is much better understood. Recent work has demonstrated that postmitotic MBs can serve a role of a polarity cue during cell differentiation. For example, several recent studies have shown that abscission is a first symmetry-breaking event that determines the location of the apical lumen during epithelial tissue morphogenesis [17,18,19]. Similarly, the MB was suggested to define the site of neurite sprouting in fly as well as to regulate the orientation of the mitotic spindle during *C. elegans* embryogenesis [20]. However, the importance of this MB-dependent mitotic spindle orientation remains controversial and requires further investigations [21].

## 4. Midbody Degradation

Since it is now well established that MBs play key roles during division as well as after completion of cytokinesis, one would expect that the inheritance and retention of postmitotic MBs is a highly regulated event. One of the ways to degrade inherited postmitotic MBs is via the autophagy. Consequently, high autophagic activity-exhibiting cells do not accumulate MBs while cells with lower autophagic activity, such as cancer stem cells, are prone to accumulate inherited postmitotic MBs [16,22]. It has been proposed that inherited intracellular postmitotic MBs can be cleared via encapsulation by the autophagic isolation membrane and eventual degradation by fusing with lysosomes to form a phagolysosome [1,16]. Consistent with this idea, it was shown that the knock-down of Atg5, the protein required for autophagy, leads to postmitotic MB accumulation as well as induces cell proliferation and anchorage independent growth. What is much less clear is how cells decide whether to keep or to degrade postmitotic MBs. It has been suggested that MB recognition and degradation relay on specific cytoplasmic receptors that tag MBs for autophagic degradation [16]. Indeed, it has been shown that NBR and p62, both known as autophagy receptors, are required for MB degradation [16]. Another way to regulate MB degradation is by affecting the extension of the isolation membrane. When the MB is targeted for degradation, the cell starts forming an isolation membrane around the MB by the recruitment of a specialized membrane structure, known as a phagophore [23]. The phagophore is formed by the recruitment of LC3 that stably associates with the phagophore membrane. Due to the large size of postmitotic MBs, the formation and extension of the autophagic isolation membrane is dependent on the delivery of additional membranes [24]. At least in part this extension is regulated by FYCO1 protein that can bind to LC3 and is also present at the phagophore [25]. A recent study has shown that FYCO1 is also required for the formation of the autophagic isolation membrane around the MB (insert in Figure 1 (7B)) [24]. Importantly, FYCO1 is downregulated in many aggressive cancers, and FYCO1 knock-down leads to MB accumulation and increase in cancer cell aggressiveness [24].

The inset in 7B shows the localization of FYCO1-mCherry in the isolation membrane of MB-containing autophagosome [24].

## 5. Conductance and Permeability of Midbody-Containing Intercellular Bridges

**Figure 2:**
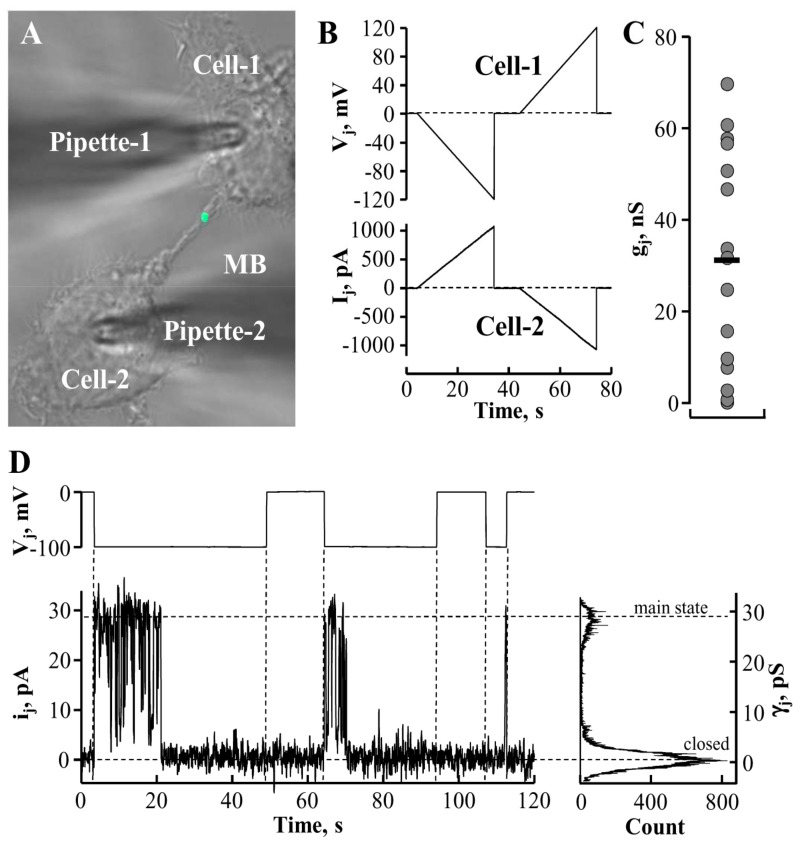
Characterization of MB-containing intercellular bridge (ICB) electrical properties. (**A**) The MB-containing ICB connecting a pair of HeLa cells expressing MKLP1-EGFP. (**B**) Electrical properties of ICBs were evaluated by applying a voltage ramp from 0 to ±120 mV (B, upper panel) to the cell-1 and measuring junctional current in the cell-2 (B, lower panel). Absence of voltage gating implies that the ICB did not contain gap junction (GJ). (**C**) Measured conductances of MB-containing ICBs (*n* = 15; the horizontal bar indicates a mean value). (**D**) Abscission may result in the formation of GJ composed of ~30 pS single channels typical of endogenous Cx45.

Gap junction (GJ) channels and membranous tunneling tubes (MTTs) or nanotubes provide a direct pathway for electrical, metabolic, and genetic communication between cells [26,27]. Membranous tunneling tubes are much longer structures than ICBs formed during cytokinesis. The permeability properties of MTTs are relatively well characterized (see, for example [26,27,28]). However, MTTs do not contain MBs. It is insufficiently addressed by now whether ICB electrical conductance as well as permeability for larger molecules is affected by the presence of the MB. To examine these possibilities, we employed fluorescence microscopy, time-lapse imaging, and dual whole-cell patch-clamp techniques [28,29]. The latter one allowed us to understand whether ICBs containing MBs (Figure 2A) preserved electrical conductance between the cells, and if so, whether these ICBs contained GJs. To test this, we measured the I_j_ response in cell-2 (Figure 2B, lower panel) to the voltage ramp from 0 to 120 mV in cell-1 (Figure 2B, upper panel). Depending on the stage of abscission, the ICBs exhibited electrical conductances (obtained from ratio ΔI_j_/ΔV_j_) from less than 1 nS to 70 nS (32 ± 6 nS; *n* = 15; Figure 2C). The absence of voltage gating suggested that these ICBs were open-ended channels not containing GJs. We assume that only the highest measured conductances represent the conductance of newly formed intact MB-containing ICBs. During the process of abscission, the conductance of the ICB ought to decrease gradually until it completely closes. Interestingly, in five more cases we measured zero conductance; however, having switched to the single-channel recording mode, in three cases we detected single channels (Figure 2D) with single-channel conductance of γ_j_ = ~30 pS, typical of connexin 45 (Cx45) [30]. HeLa cells express low levels of endogenous Cx45 that may form low transjunctional conductance GJs [31]. Single-channel currents were measured in the cell-2 as a response to voltage step of −80 mV in the cell-1, and γ_j_ was obtained from ratio i_j_/V_j_. This observation suggests that at the end of cytokinesis, electrical coupling between cells may be preserved by forming GJs between them. Currently we cannot suggest other than abscission site for GJ channel formation and, unfortunately, immunolabeling cannot help to double-check the location of a single channel. We expect to address this question in our further investigations employing other types of cells capable of forming functional high-conductance GJs.

**Figure 3:**
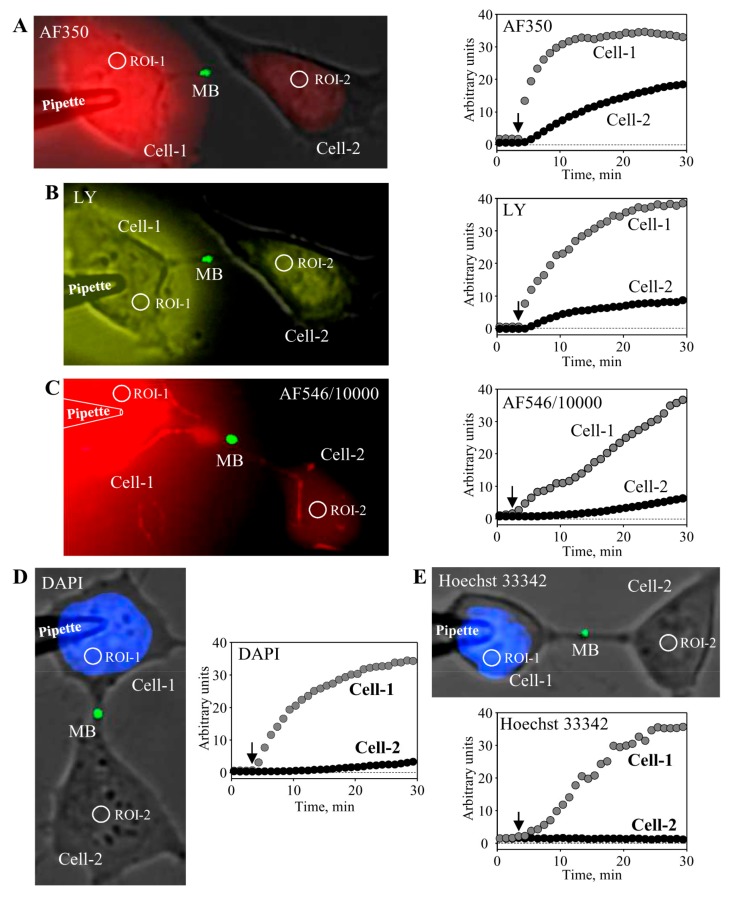
Characterization of MB-containing ICB permeability. To measure the permeability of MB-containing ICB, HeLa cells expressing MKLP1-EGFP were used. The pipette filled with the dye was attached to the cell-1. After opening patch by electrical pulse, the dye diffused to the cell-1 followed by dye transfer through the ICB to the cell-2. Kinetics of dye accumulation in both cells was measured by time-lapse imaging. Cells were exposed to low-intensity light for ~0.5 s every 1 min to minimize dye bleaching. During the entire experiment, tested cells were maintained in continuous external perfusion through a rectangular glass capillary situated in the proximity to the cells in order to prevent possible external contamination of cells with the dye due to its leak from the patch pipette. Representative images from at least three independent experiments for each fluorescent dye are shown. MB-containing ICBs were permeable to negatively charged dyes AF350 (A, 1 mM), LY (B, 1 mM), and AF546/10,000 (C, 0.1 mM) and impermeable to positively charged dyes DAPI (D, 0.1 mM) and Hoechst-33342 (E, 0.05 mM).

Further, we examined the permeability of MB-containing ICBs to fluorescent dyes of different molecular weight and net charge. We used the following dyes (molecular mass of the fluorescent ion; valence): Alexa Fluor-350 (AF350) (326; −1), Lucifer Yellow (LY) (443; −2), Alexa Fluor-546/10,000 dextran (AF546/10,000) (10,000; −1), DAPI (279; +2), and Hoechst-33342 (562; +3). To measure the permeability of the MB-containing ICB, the pipette filled with the dye was attached to the cell-1 (Figure 3). After opening patch by electrical pulse, the dye diffused to the cell-1 followed by dye transfer through the ICB to the cell-2. Kinetics of dye accumulation in both cells is shown in the respective panels of Figure 3. These results demonstrate that MB-containing ICBs were permeable to dyes of negative net charge such as AF350 (Figure 3A) and LY (Figure 3B), as well as even such a large molecule as AF546/10,000 (Figure 3C). However, ICBs were virtually impermeable to dyes of positive net charge such as DAPI (Figure 3D) and Hoechst-33342. At the end of the experiment, the patch in the cell-2 was opened with the second pipette to measure electrical conductance of ICBs in a dual whole-cell patch-clamp mode. These results indicate that MBs may play a role of selectivity filter for positively charged molecules, because tunneling nanotubes that do not contain MBs allowed the passage of molecules independent of their charge polarity, but rather dependent on charge size [28]. Gap junctions allow the passage of molecules up to ~1 kDa, such as cAMP, ATP, ADP, AMP, adenosine, IP_3_, glutamate, glutathione, etc. (reviewed in Reference [27]). Family of connexins (Cxs) composing GJ channels consists of 21 genes in the human genome. Some of them are more permeable to positively charged, others to negatively charged molecules, and in general, GJ permeability is inversely dependent on charge size [32]. Low concentrations of Ca^2+^ can permeate GJ channels; however, higher concentrations close the channels directly or in calmodulin-dependent manner [33]. Mg^2+^ and H^2+^ also gate GJs in concentration- and Cx type-dependent manner [34,35].

Our observations that GJ channels appear between daughter cells at the end of ICB abscission and that MB-containing ICBs have a strong preference for negatively charged molecules suggest that one of the properties of MBs might be to play a GJ role in attenuating Ca^2+^, Mg^2+^, and H^+^ fluctuations between cells until they establish GJ-dependent communication. However, further studies are required to verify this hypothesis and understand its physiological significance.

## 6. Concluding Remarks

Midbody inheritance and accumulation in cells lead to reprogramming of cell fate and conversion to highly proliferative stem cell-like phenotypes. However, the mechanisms that regulate asymmetric MB inheritance and post-mitotic degradation remain completely unknown. Proteomic analysis of the MB identified hundreds of proteins pointing out that this organelle has powerful and sophisticated machinery capable of regulating the fate of stem cells and cancer stem cells. Therefore, the major future challenges should include the determination of putative proteins or protein complexes related to specific cellular mechanisms regulating their proliferation, differentiation, development, intra- and intercellular communication, tumorigenicity, and other properties.

## Supplementary Materials

The following are available online at http://www.mdpi.com/1010-660X/54/4/53/s1, Video S1: Midbody formation; Video S2: Midbody inheritance after asymmetric abscission; Video S3: Midbody release into the extracellular space after symmetric abscission.
